# The Idea of Progress

**Published:** 1920-06-05

**Authors:** 


### THE PUBLIC WELL-BEING.

### The Idea of Progress.

A question of great interest was raised by the Dean of St. Paul's in his Romanes lecture at Oxford last week, when he challenged the general belief in progress, not as a task for humanity, but as a law of Nature. This challenge is not so startling as the Dean's illustrations in support of it. The idea of progress as a natural law has invaded science, religion, philosophy, and history, but if the idea is tested closely (says the Dean) it will be seen that physically the race has made no progress for thousands of years, that mentally it cannot be claimed we are the equals of the Athenians or superior to the Romans, and that the evidence of the war seems to show we are not more humane, juster, or less brutal than the ancients. The accumulated experience of mankind is of great value, but "does not constitute real progress in human nature itself."

The challenge is perhaps more stimulating than otherwise, but the arguments brought forward by the Dean in support are altogether on a different plane. If it is human effort alone that brings progress; if the facile idea that all will be well, with or without effort, is dispelled; if the saying that "God helps those who help themselves" is the true and actual philosophy when taken in the broadest sense, then let us stiffen the sinews and brace the will. Progress is in our keeping, and what we sow we reap. There is reality and there is hope. But if we have not progressed for thousands of years except in the accumulation of experience, what then? Surely that is a hopeless outlook, and one to which it is hard to consent.

It is not easy precisely to define progress; it depends on what is the ideal goal, and this varies individually, as well as nationally. But there are broad lines generally accepted, such as greater opportunities, greater sympathies, wider political and personal freedom, a better standard of life, a greater care for the helpless, and so forth. It may be difficult to deny that the Athenians—some of them—were of better physique, and even of better culture, than the race of to-day. The Dean may be right that the prehistoric races which banged each other's heads with clubs, as we slap a friend on the shoulder, were just as good as we are. It is never easy to compare different civilisations. But were the average Athenians in the enjoyment of better corporate life, or possessed of better average culture? The ancient Greek civilisation was based on slavery, which does not exist to-day, and on this one phase alone—the abolition of the slave system—there has thus been progress, widespread in its ramifications. The comparison should not be between Socrates and a trade unionist, but between a trade unionist and a slave. For one finely-developed individual among the ancients there are a thousand now. It is much to be questioned whether the idea that our modern wars are as brutal as the ancients' would bear detailed examination. They are certainly not so numerous; there are recognised rules of warfare which did not then exist; and, though human passions may not have changed, the control of human passions may have become stronger. Despite the war, and its consequences, international ideals do exist as they did not exist in times past; the sense of justice is deeper; compassion for the unfortunate and the suffering is more marked; education is infinitely wider, with the added mental exercise and enjoyment; and individual freedom is incomparably greater. Can these things be—if so they are—without the progress of individual human nature as reflected in the progress of the race? We plump instinctively for progress through the ages, and regard hope as eternal, but not in the sense of eternally eluding our grasp. The Dean's endeavour to separate progress from "accumulated experience" is somewhat analogous to the materialist's argument that every human action is governed by the laws of heredity and environment. Everything can be conveniently explained to support the broad thesis without proving it. But accumulated experience *is* progress, and the immense category of progress under that head alone is sufficient to swamp those who would have us put away all hope and take faith only in the days that have gone.

